# Bone structure determined by HR-MDCT does not correlate with micro-CT of lumbar vertebral biopsies: a prospective cross-sectional human in vivo study

**DOI:** 10.1186/s13018-020-01895-0

**Published:** 2020-09-10

**Authors:** Matthias Pumberger, Ahi Sema Issever, Torsten Diekhoff, Christin Schwemmer, Susanne Berg, Yannick Palmowski, Michael Putzier

**Affiliations:** 1 Spine Department, Center for Musculoskeletal Surgery, Charité University Medicine Berlin, Chariteplatz 1, 10117 Berlin, Germany; 2 Department of Radiology, Charité University Medicine Berlin, Chariteplatz 1, 10117 Berlin, Germany; 3 Charité University Medicine Berlin, Chariteplatz 1, 10117 Berlin, Germany

**Keywords:** Spine, Vertebral biopsies, MDCT, Micro-CT, Osteoporosis

## Abstract

**Background:**

Osteoporosis is characterized by a deterioration of bone structure and quantity that leads to an increased risk of fractures. The primary diagnostic tool for the assessment of the bone quality is currently the dual-energy X-ray absorptiometry (DXA), which however only measures bone quantity. High-resolution multidetector computed tomography (HR-MDCT) offers an alternative approach to assess bone structure, but still lacks evidence for its validity in vivo. The objective of this study was to assess the validity of HR-MDCT for the evaluation of bone architecture in the lumbar spine.

**Methods:**

We conducted a prospective cross-sectional study to compare the results of preoperative lumbar HR-MDCT scans with those from microcomputed tomography (μCT) analysis of transpedicular vertebral body biopsies. For this purpose, we included patients undergoing spinal surgery in our orthopedic department. Each patient underwent preoperative HR-MDCT scanning (L1-L4). Intraoperatively, transpedicular biopsies were obtained from intact vertebrae. Micro-CT analysis of these biopsies was used as a reference method to assess the actual bone architecture. HR-MDCT results were statistically analyzed regarding the correlation with results from μCT.

**Results:**

Thirty-four patients with a mean age of 69.09 years (± 10.07) were included in the study. There was no significant correlation for any of the parameters (bone volume/total volume, trabecular separation, trabecular thickness) between μCT and HR-MDCT (bone volume/total volume: *r* = − 0.026 and *p* = 0.872; trabecular thickness: *r* = 0.074 and *r* = 6.42; and trabecular separation: *r* = − 0.18 and *p* = 0.254).

**Conclusion:**

To our knowledge, this is the first study comparing in vivo HR-MDCT with μCT analysis of vertebral biopsies in human patients. Our findings suggest that lumbar HR-MDCT is not valid for the in vivo evaluation of bone architecture in the lumbar spine. New diagnostic tools for the evaluation of osteoporosis and preoperative orthopedic planning are urgently needed.

## Introduction

Osteoporosis is a chronic metabolic bone disease that leads to an increased risk of fractures due to a deterioration of bone structure and quantity [1]. With an estimated number of 2.7 million osteoporotic fractures per year in Europe alone, causing direct costs of €36 billion per year, osteoporosis constitutes a major medical and socioeconomic challenge—especially given its continuously increasing prevalence in aging societies [2]. Yet, the relevance of osteoporosis is not limited to the increased risk of fractures. Reliable knowledge about local bone quality is also of high importance in the surgical field as it is indispensable for the planning of any orthopedic surgery. In spinal surgery, a compromised bone architecture increases the risk of complications and may require adaptations of the surgical proceeding [3–5].

Regarding the diagnosis of osteoporosis, current guidelines recommend a two-step proceeding. The first step should always be a systematic patient history for an evaluation of relevant risk factors [6, 7]. If an increased risk of fractures has to be suspected, dual-energy X-ray absorptiometry (DXA) measurement is the current radiological standard to confirm the diagnosis [6, 7]. Additionally, DXA also remains the most important tool for monitoring purposes, e.g., to evaluate the response to antiresorptive medications during follow-ups, and for preoperative bone assessment [7–9].

However, various concerns have recently been raised questioning its validity for the abovementioned purposes. To start with, DXA is only able to measure bone density. However, bone density can only explain about 60–70% of total bone strength [10]. The structure, which seems to be another important factor, is not accounted for. Further problems arise from the fact that DXA can only measure a 2D projection of the density that includes overlapping structures. In the lumbar spine, there are particular concerns about the influence of unavoidable intra- and extra-osseous soft tissue contributions [11]. Mineral in the facet joints and the aorta caused by degenerative changes may lead to artificially increased results in elderly patients [12, 13]. This skepticism about the use of DXA is even further aggravated by its high precision error, with only changes of more than 5% being significant in lumbar DXA [14].

These findings result in an ongoing need for alternative approaches to enable a reliable evaluation of the bone quality, particularly in the lumbar spine. One potential substitute is the high-resolution multidetector computed tomography (HR-MDCT), which offers several advantages over DXA. With 3D image acquisition and voxel sizes of ≤ 1 mm, it delivers a detailed impression of the bone structure, which is not captured by DXA. Besides, this also allows a more precise positioning of the region of interest (ROI) to reduce the influence of soft tissues or sclerotic changes. Another advantage of the precise and site-specific evaluation of bone properties is the potential usefulness for orthopedic planning, e.g., to assess the morpho-densitometric characteristics of lumbar pedicles before spinal surgery or for intertrochanteric femoral fractures [15, 16]. The possibility to use regular MDCT scanners instead of dedicated machines results in a far higher availability. Numerous cadaveric and animal studies have already shown promising results, e.g., a correlation of MDCT-derived bone parameters with biopsies examined by μCT or direct biomechanical testing [17–21]. Some articles have indicated that even scans performed for other reasons like MDCT colonoscopy can be analyzed in a way that allows assumptions on bone quality [22–26].

However, evidence on the in vivo application in human patients is still scarce, limiting it primarily to research applications for the time being [27]. To our knowledge, no study has so far examined the validity of spinal MDCT to represent actual bone architecture. We hypothesized that high-resolution MDCT might be an adequate tool for the diagnostic evaluation of bone architecture and designed a prospective controlled study to evaluate MDCT measurements in correlation to μCT analysis of vertebral biopsies, which is considered the gold standard for assessing bone above the cellular level [28].

## Materials and methods

### Patients

For this prospective cross-sectional study, we included patients that were scheduled for spinal surgery in our department for orthopedics. Patients were recruited between October 2012 and November 2014 at our university orthopedic outpatient department. Exclusion criteria included the diagnosis of secondary osteoporosis, anti-osteoporotic medication, history of previous tumor or autoimmune disease, previous surgery of the respective vertebrae, or patients unable to give informed consent.

### High-resolution multidetector computed tomography (HR-MDCT)

**Fig. 1:**
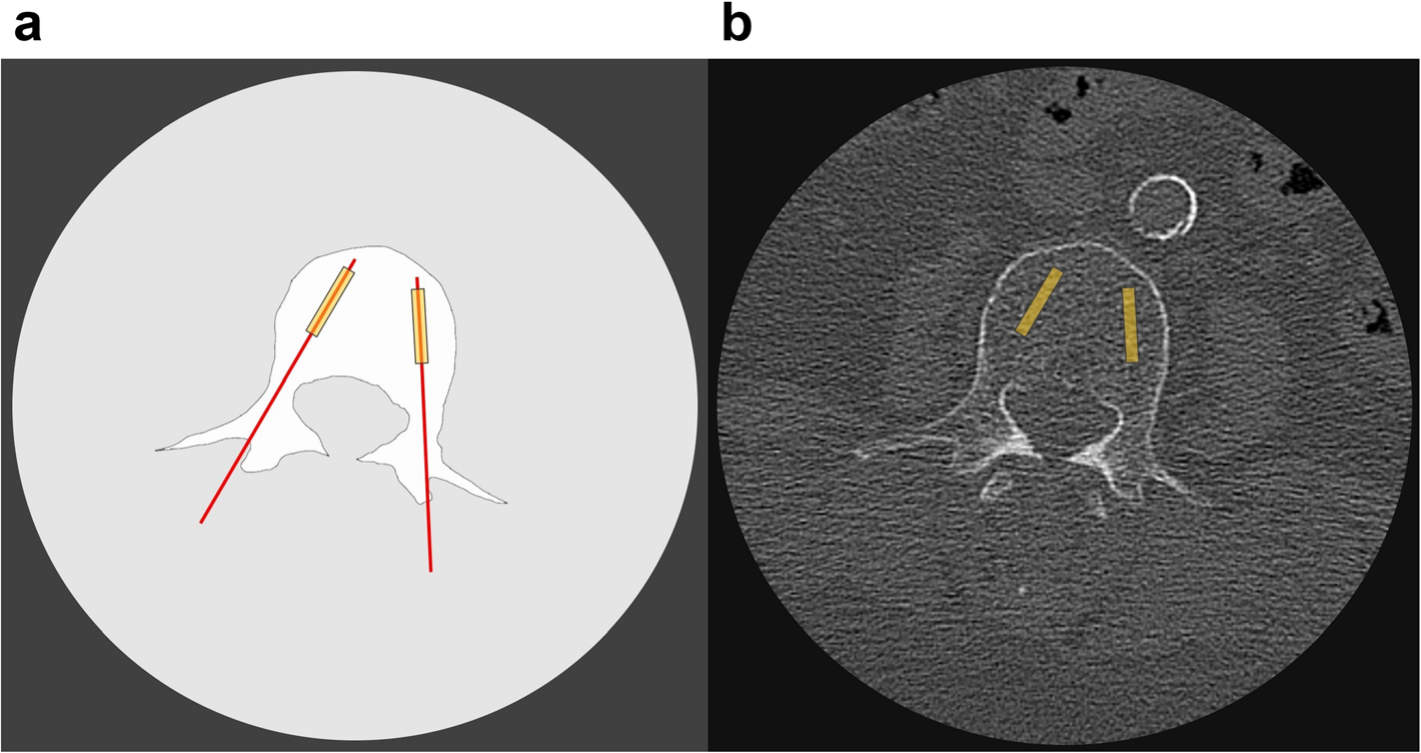
**a** Acquisition of biopsies: the line depicts the transpedicular access path to the vertebral body, the box depicts the area of biopsy acquisition. **b** Structure analysis using HR-MDCT: the box depicts the position of the region of interest (ROI) in HR-MDCT

Prior to the respective intervention, all patients received an HR-MDCT of the lumbar spine in a supine position without intravenous contrast administration. The scan was performed with an Aquilion 64 machine (Canon Medical Systems, former Toshiba) with a tube current of 100 mAs, 0.5 s rotation time, and a tube voltage of 120 kVp. This results in a CTDIvol of 12.24 mGy. A standard bone kernel (FC 30) was reconstructed using filtered back projection with a slice thickness of 0.5 mm and 0.5 mm spacing resulting in 0.5 mm^3^ isotropic voxel, the highest special resolution for MDCT available at that time. A region of interest (ROI) was placed on the images in the presumed area of the biopsy (Fig. 1). The structural parameters bone volume/total volume (BV/TV), trabecular thickness (Tb.Th), and trabecular separation (Tb.Sp) were analyzed.

### Operation and acquisition of specimens

A transpedicular biopsy from the cancellous bone was harvested in all patients from an intact vertebral body. For this purpose, L3 was chosen as the reference vertebral body. In cases where a biopsy from L3 was not possible, e.g., due to a vertebral fracture or previous surgery, the biopsy was obtained from one of the adjacent vertebrae (preferable L4, or alternatively L2) instead. From each vertebral body, two biopsies were obtained: one through the left and one through the right pedicle.

The same technique was used to harvest all biopsies. The patient was placed in a prone and true anterior-posterior position. Using the lateral projection of a C-Arm, it was confirmed that the trocar was placed directly at the boarder of the pedicle. All biopsies were taken from the full length of the vertebral body using a Jamshidi bone biopsy needle (Allegiance Healthcare, Unterschleißheim, Germany) and subsequently processed in an identical manner. They were fixated for 48 h in a 4% formaldehyde solution, then watered for 20 min and kept in a phosphate buffer solution until further processing.

### μCT

After careful placement in a plastic pipette filled with phosphate-buffered saline, all specimens were examined in a vivaCT 40 μCT device (Scanco Medical, Brüttisellen). Using a scout view, two preferably homogenous and intact target areas were chosen. Both were measured in 95 slices each with a voxel size of 10.5 μm comprising the whole circumference of the sample, resulting in two scanning areas with a length of 997.5 μm each. Each slice was outlined half-automatically in order to define the ROI. Histograms were generated and used to calculate the threshold of 308 mg HA/cc, which was applied to all specimens. Furthermore, 3D reconstructions of each biopsy were evaluated independently by two members regarding the bone integrity and graded from 1 (very good interpretability) to 3 (reduced interpretability). All specimens classified as grade 3 were excluded from statistical analysis in order to avoid a corruption of the results due to bone damages from the acquisition of the biopsy. The results of both target areas were calculated into a mean score for each biopsy. Technical details were described previously [29].

### Statistical analysis

Descriptive analysis including mean values and standard deviation was performed. For the comparison of μCT and HR-MDCT, the μCT results of each biopsy were juxtaposed with the results of the respective ROI from HR-MDCT (left or right) and the Pearson’s correlation coefficient was calculated for comparative statistical analysis.

## Results

### Patients and samples

**Fig. 2:**
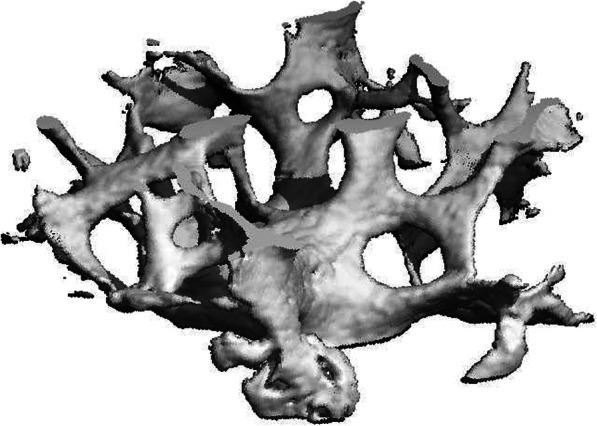
3D reconstruction of a vertebral biopsy

A total of 34 patients (22 women, 12 men) were included in this study. The mean age was 69.09 (± 10.07). In 29 cases, the biopsies were obtained from L3, in two cases from L2 and in three cases from L4. In four patients, no biopsy of adequate quality for reliable μCT analysis could be obtained. Therefore, 30 patients were included for statistical analysis. An example of the 3D reconstruction of an adequate biopsy is depicted in Fig. 2.

### Correlation HR-MDCT and μCT

**Fig. 3:**
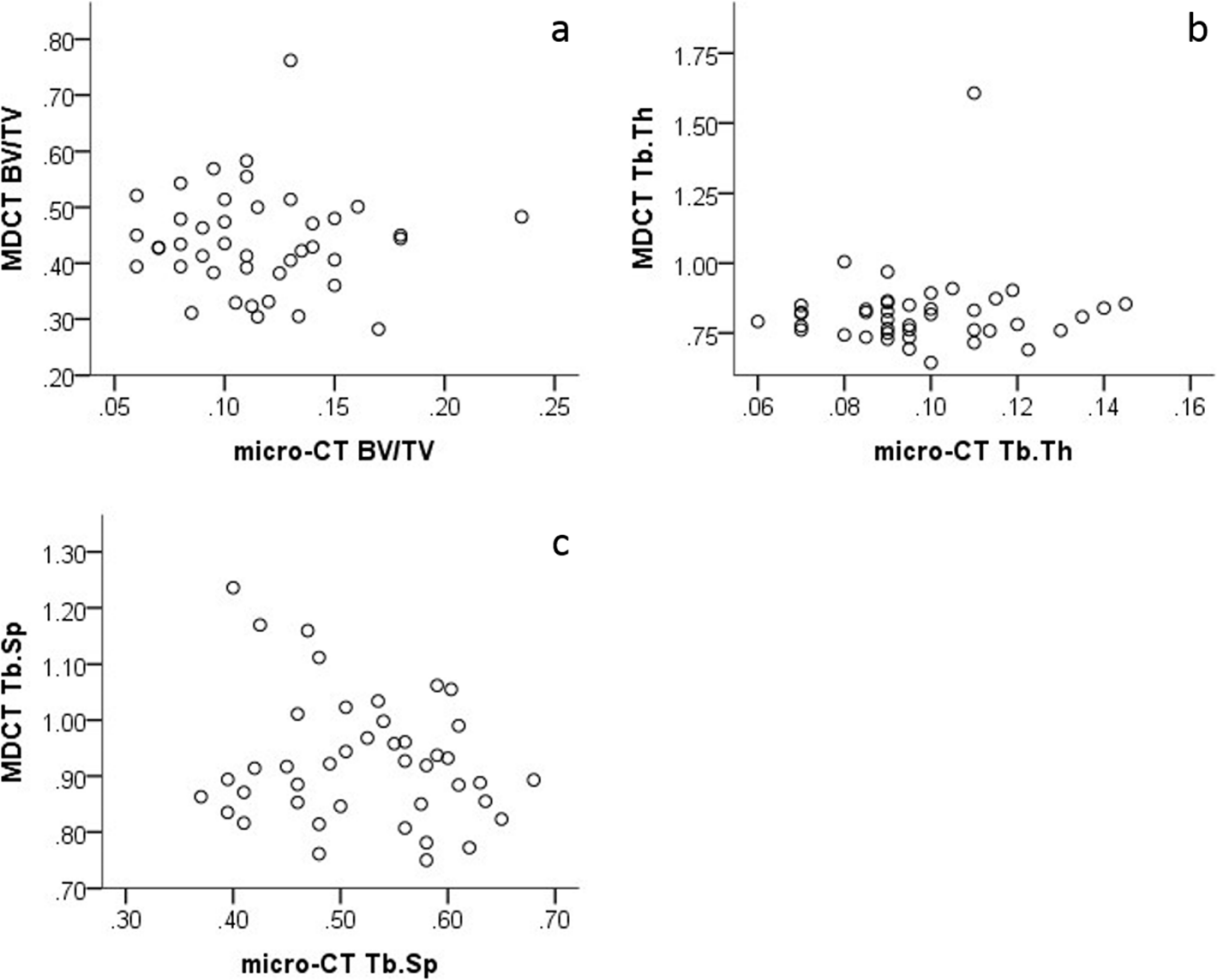
**a** Scatter plot of bone volume/total volume in μCT of vertebral biopsies and in vivo HR-MDCT of the same vertebrae. **b** Scatter plot of trabecular thickness in μCT of vertebral biopsies and in vivo HR-MDCT of the same vertebrae. **c** Scatter plot of trabecular separation in μCT of vertebral biopsies and in vivo HR-MDCT of the same vertebrae

There was no significant correlation for any of the examined parameters between HR-MDCT and μCT—neither for quantitative (BV/TV), nor for qualitative (Tb.Th, Tb.Sp) parameters (Table 1, Fig. 3).

**Table 1:** Correlation of HR-MDCT and μCT results.

Pearson’s correlation coefficient (***r***) and ***P*** value (***p***)				
		μCT BV/TV	μCT Tb.Th	μCT Tb.Sp
MDCT BV/TV	*r*	− .026	− .068	− .014
	*p*	.872	.669	.929
MDCT Tb.Th	*r*	.046	.074	− .060
	*p*	.774	.642	.707
MDCT Tb.Sp	*r*	.020	.082	− .180
	*p*	.899	.606	.254

## Discussion

The present study constitutes the first comparison of in vivo HR-MDCT with the actual bone structure of vertebral biopsies. Whereas previous ex vivo studies showed promising results, we found no significant correlation of the structural parameters as measured in HR-MDCT and μCT [20].

We used μCT as a reference method to determine the actual bone architecture. μCT is a highly precise and reliable procedure for the in vitro evaluation of three-dimensional trabecular bone structure and is considered the gold standard for assessing bone above the cellular level [28, 30, 31]. It has shown good sensitivity to monitor postmenopausal osteoporotic changes in animal studies [32] and a higher validity than DXA for the evaluation of mechanical bone properties in ex vivo studies [33–35]. However, there is one major downside preventing a widespread application in clinical practice: the inevitable invasiveness to obtain the necessary biopsies. In order to circumvent this hindrance, we only included patients that were scheduled for an operation on the lumbar spine independently from the acquisition of the biopsies. Also, preoperative planning required a CT scan for the included patients in any way, so that they were not exposed to any additional radiation either. Thereby, we prevented any additional harm resulting from the participation in this study. At the same time, this study design helped to ensure the applicability of our results to relevant populations, as reliable knowledge about the bone quality is of particular importance in patients undergoing spinal surgery.

Another problem related to the biopsies needed for μCT is the risk of damaging the specimens during the acquisition. In order to avoid a corruption of the results, 3D reconstructions of each μCT measurement were independently rated by two members regarding the integrity of the bone architecture and those with a reduced rating were excluded from statistical analysis. For the same reason, i.e., to avoid a negative influence on the results due to flawed measurements of the damaged bone, we only analyzed biopsies taken from intact reference vertebrae in this study and did not evaluate osteoporotic vertebrae that had already fractured.

Several cadaveric studies have shown a correlation between μCT and HR-MDCT, which could not be confirmed by the present study [36, 37]. The comparison between the structural results of μCT and HR-MDCT did not show any significant correlation between the two methods—neither with qualitative, nor quantitative parameters. This is particularly remarkable as both methods rely largely on the same principles and can be used to measure exactly the same parameters. The main handicaps of HR-MDCT are the lower resolution (slice thickness of 500 μm as compared to 10.5 μm in our study) and the influence of surrounding soft tissues, which also poses a problem to other radiologic methods like DXA [11]. It must be noted that most of the cadaveric studies that found a significant correlation were either carried out in vitro or on peripheral bones surrounded by less soft tissue, like the humerus or ankle. Apparently, the results of these studies cannot be transferred to the spine in vivo.

Another potential reason for the differences between both approaches is the part of the bone that is measured. The HR-MDCT is able to capture the whole vertebrae, whereas the μCT is always limited to a small biopsy. Therefore, the inclusion of areas in HR-MDCT that are not comprised in the biopsy might lead to different results. However, we took account of this issue by restricting the ROI of the HR-MDCT measurements to the region where the biopsies were to be taken (Fig. 1) in order to guarantee optimal comparability. Still, it is possible that the chosen ROI did not always completely match the area of the biopsy. A further factor likely to contribute to the difference in measurements between methods is the partial volume effect. This effect occurs if an object is smaller than the voxel that depicts it or only extends into it. As a consequence, the object, e.g., a bone trabecula, only constitutes a part of the whole voxel and the depicted density is the mean value from the object itself and the remaining tissues contained in the voxel. This effect has already been described as a cause for over- or underestimation of certain parameters in animal studies [28].

Yet, it needs to be taken into account that the results of this study only represent a rather small local patient collective. Additional studies will be needed to confirm our findings in larger populations. Furthermore, we did not conduct a longitudinal observation. Therefore, no conclusion can be made concerning the predictive value of parameters derived from HR-MDCT or μCT for material failure or future fractures.

In conclusion, the findings of our study suggest that HR-MDCT—unlike previously reported—is no valid tool for the in vivo evaluation of bone architecture in the lumbar spine, neither qualitatively nor quantitatively. Therefore, it is no appropriate technique to replace DXA for the prediction of vertebral fractures or preoperative orthopedic planning. This poses an important problem as DXA itself has already raised serious concerns about its validity in the spine, too. Thus, new methods for the in vivo evaluation of the bone quality in the spine are urgently needed and existing alternatives need careful evaluation.

## Data Availability

The datasets used and/or analyzed during the current study are available from the corresponding author on reasonable request.
